# Prolonged Mechanical Ventilation and Extubation Failure in Pediatric Patients Undergoing Surgical Correction for Congenital Heart Disease

**DOI:** 10.21470/1678-9741-2025-0228

**Published:** 2026-05-06

**Authors:** Felipe Borsu de Salles, Paulo Eduardo Meneguzzo

**Affiliations:** 1 Cardiac Surgery Department, Hospital Divina, Porto Alegre, Rio Grande do Sul, Brazil E-mail: sallesfb@gmail.com; 2 Cardiac Surgery Department, Hospital Moinhos de Vento, Porto Alegre, Rio Grande do Sul, Brazil

Dear Editor,

Costa et al.^[1]^ conducted a valuable retrospective cohort study evaluating the association of clinical and biological factors with prolonged mechanical ventilation (PMV) and extubation failure (EF) in pediatric patients undergoing surgical correction for congenital heart disease (CHD). A variety of preoperative clinical variables were collected and analyzed to identify potential risk factors for PMV and EF. The study is valuable due to its large sample size from a nationally relevant tertiary center.

Although the study was designed retrospectively beginning in March 2020, patient inclusion continued through December 2021. This overlap with ongoing surgical care may introduce a Hawthorne effect, potentially influencing both data recording and clinical management where awareness of ongoing evaluation could influence clinical behavior or data entry, potentially biasing key outcomes like sedation strategy or extubation timing. A prospective database could help standardize data collection and support the implementation of care protocols, offering a more accurate representation of institutional practices and aiding future decision-making.

Key variables such as *extubation failure* and *spontaneous breathing trial* should be clearly defined, as definitions often vary across studies^[2]^. Additionally, the authors could elaborate on their rationale for the definition of PMV used. Notably, more than 50% of PMV-related studies do not explain their chosen definition^[3]^, which limits comparability and reproducibility.

Furthermore, future analyses should consider additional clinical variables known to influence the duration of mechanical ventilation. These include the presence of congenital anomalies in other organ systems, scoliosis or thoracic deformities, chromosomal abnormalities or genetic syndromes, the need for vasoactive support, and the rationale for maintaining continuous sedation in the immediate postoperative period - whether due to respiratory, cardiac, neurological, or infectious conditions.

A previous Brazilian study^[4]^ demonstrated that the duration of postoperative mechanical ventilation in cardiac surgery patients is directly related to the complexity of the underlying cardiac defect, a finding consistent with international literature^[5]^.

Further exploration of the subgroup with PMV and EF could yield valuable insights, particularly regarding the type of CHD, fluid and intraoperative management, intensive care unit and hospital length of stay, and associated morbidity and mortality.

Sincerely,

## Artificial Intelligence Usage

The authors declare use of free version of ChatGPT-4o for grammatical correction of the English version of this text.
